# Enhancing Europium Adsorption Effect of Fe on Several Geological Materials by Applying XANES, EXAFS, and Wavelet Transform Techniques

**DOI:** 10.3390/toxics12100706

**Published:** 2024-09-28

**Authors:** Chi-Wen Hsieh, Zih-Shiuan Chiou, Chuan-Pin Lee, Shih-Chin Tsai, Wei-Hsiang Tseng, Yu-Hung Wang, Yi-Ting Chen, Chein-Hsieng Kuo, Hui-Min Chiu

**Affiliations:** 1 Department of Electrical Engineering, National Chung Cheng University, Chiayi County 621301, Taiwan; chiwenh@ccu.edu.tw (C.-W.H.); a067213121@gmail.com (Z.-S.C.); hot0213338@gmail.com (W.-H.T.); 2 Center for Energy and Environmental Research, National Tsing Hua University, Hsinchu City 300044, Taiwan; 3 Radioactive Waste Disposal Technology Research and Development Center, National Tsing Hua University, Hsinchu City 300044, Taiwan; henry911219@gmail.com (Y.-H.W.); cyiting000@gmail.com (Y.-T.C.); lspss97242@gmail.com (C.-H.K.); mickly1212@gmail.com (H.-M.C.); 4 Nuclear Science and Technology Development Center, National Tsing Hua University, Hsinchu City 300044, Taiwan; 5 Department of Mathematics, National Tsing Hua University, Hsinchu City 300044, Taiwan; 6 Department of Quantitative Finance, National Tsing Hua University, Hsinchu City 300044, Taiwan; 7 Department of Industrial Engineering and Engineering Management, National Tsing Hua University, Hsinchu City 300044, Taiwan; 8 Department of Environmental Engineering and Health, Yuanpei University of Medical Technology, Hsinchu City 300102, Taiwan

**Keywords:** argillite, basalt, granite, biotite, batch tests, X-ray, wavelet-transform

## Abstract

This study conducted adsorption experiments using Europium (Eu(III)) on geological materials collected from Taiwan. Batch tests on argillite, basalt, granite, and biotite showed that argillite and basalt exhibited strong adsorption reactions with Eu. X-ray diffraction (XRD) analysis also clearly indicated differences before and after adsorption. By combining X-ray absorption near-edge structure (XANES), extended X-ray absorption fine structure (EXAFS), and wavelet transform (WT) analyses, we observed that the Fe_2_O_3_ content significantly affects the Eu-Fe distance in the inner-sphere layer during the Eu adsorption process. The wavelet transform analysis for two-dimensional information helps differentiate two distances of Eu-O, which are difficult to analyze, with hydrated outer-sphere Eu-O distances ranging from 2.42 to 2.52 Å and inner-sphere Eu-O distances from 2.27 to 2.32 Å. The EXAFS results for Fe_2_O_3_ and SiO_2_ in argillite and basalt reveal different adsorption mechanisms. Fe_2_O_3_ exhibits inner-sphere surface complexation in the order of basalt, argillite, and granite, **while** SiO_2_ forms outer-sphere ion exchange with basalt and argillite. Wavelet transform analysis also highlights the differences among these materials.

## 1. Introduction

Achieving net-zero carbon emissions is a current goal for countries worldwide, making nuclear energy an essential and indispensable energy source. The final disposal of high-level nuclear waste has become a critical issue. Nuclear waste is categorized based on its radioactivity levels, with high-level nuclear waste (HLW) being the most dangerous due to its long-lived radioisotopes. In Taiwan, the disposal of nuclear waste has been a contentious issue, with the country currently storing spent nuclear fuel and other radioactive waste at temporary facilities. The geological conditions in Taiwan pose unique challenges for the HLW, making it essential to understand the interactions between radioactive nuclides and local geological materials [1]. To assess its potential pollution to the natural environment, the analysis of the adsorption mechanism of radioactive nuclides in natural materials is significant, especially when conducting comparative studies on Taiwan’s local geological materials and common minerals. This will be immensely helpful in determining the treatment and disposal sites for high-level nuclear waste. Previous studies have investigated the removal of Cs and Sr from contaminated water using bentonite–alginate MCs in ZH, GMZ, and MX80 bentonite [2]. The diffusion behavior of Se(IV) in Tamsui Mudrock was studied in terms of pH value, ionic strength, and humic acid (HA) through diffusion methods [3]. Adsorption experiments applied two heterogeneous isotherm models to study the adsorption characteristics of Nb on Kinmen granite, mudstone, and MX-80 bentonite [4]. These studies and methods help us understand the adsorption mechanisms of radionuclides in the environment to assess the safe disposal of high-level radioactive waste in Taiwan.

Since Eu(III) is considered a chemical analog of the long half-life and radiotoxic trivalent lanthanides Am(III) and Cm(III), there has been extensive research on the adsorption of Eu(III) on metal oxides, minerals, and clay materials, such as hematite [5], muscovite [6], titanium dioxide [7,8,9], smectite [10], kaolinite [10], and granite [11]. Studies have found that the adsorption of Eu(III) is closely related to pH, with adsorption increasing as the pH value rises. This shift is driven by a transition from ion exchange mechanisms to surface complexation mechanisms [7,8]. This is particularly important when using X-ray absorption spectroscopy, as it can provide information about the adsorption mechanisms occurring around atomic structures, such as explanations of the Eu-O distances and their coordination numbers on hydrated and mineral surfaces. This helps us understand the microscopic interactions between these geological materials and radionuclides.

In this work, we combined various analytical methods to investigate the adsorption mechanism of Eu(III) on different Taiwanese geological materials, with a particular focus on the atomic structure of inner-sphere complexes. The integrated data suggest that the adsorption mechanism of Eu(III) is influenced by Fe, which causes the adsorption sites to shift closer to the mineral’s inner layer. The application of wavelet transformation enhances the observation of this phenomenon and provides an important reference for EXAFS analysis.

## 2. Materials and Methods

### 2.1. Rock Materials and Solid Phase Analysis

Argillite samples were collected from the Dazen Township of Taitung County at an outcrop along the Fongkang River. Basalt was drilled and collected from Penghu Islands, located at latitudes and longitudes of 23° N and 119° E, respectively. granite rock core samples were collected from the Wuchiu discrete islands of Kinmen by the Industrial Technology Research Institute (ITRI), Taiwan. Biotite was adopted from POWCHUNG ENTERPRISE CO.’s rock-forming mineral specimens, with the company located in Taipei City 106032, Taiwan.

In this study, both a wavelength dispersive X-ray fluorescence (WDXRF) spectrometer (Axios, PANalytical Inc., Amsterdam, The Netherlands) and the interlayer distance changes for Eu adsorbed on several rocks, including basalt, argillite, granite, etc., at room temperature were obtained by X-ray diffraction (XRD, BL17A, NSRRC, Hsinchu City 300091, Taiwan) on beamline 17A at the National Synchrotron Radiation Research Center (NSRRC) in Taiwan. The chemical compositions of these samples are given in Table 1, and there are about 60–70% alumina/silicates (Al/Si oxides) minerals in three rocks. These mineral components and interlayer distance changes were analyzed and compared for Eu adsorption by XRD analysis.

### 2.2. Batch Tests

The batch test method [12] was considered a suitable method to study the reaction of Eu in disposal sites. Thus, a simulation of the sorption experiments for different host rocks or buffer materials was performed using a batch test. Distribution of Eu in the host rock or buffer materials was observed by measuring the pH, Eh, and the final concentration. The rocks selected for the batch test were crushed in a grinder and the particles were passed through a 200-mesh sieve (<0.074 mm). Furthermore, to ensure the reliability of the experimental data, we conducted triplicate experiments and included a blank control (C₀) to verify the reproducibility of each dataset.

The batch test method reported by ASTM was adopted in this study for rock samples. Thus, a 1 g sample and the host rock and bentonite in a fixed proportion (1 g:30 mL) were placed in a 50 mL centrifuge tube, respectively. The initial concentration (C₀) of Eu was prepared and analyzed to be 10 ppm, using Eu_2_O_3_ dissolved in a 1 M NO_3_^−^ solution. The centrifuge tube was placed in a thermostatic shaker and uniformly mixed using oscillation (200 rpm) for 7 days. The solid and liquid phases were separated by the high-speed centrifuge (=10,380× *g*, Kokusan H-200, Tokyo, Japan) for 30 min. In addition, its pH and Eh values were also measured by glass electrode (InoLab-412, Mettler Teledo, Greifensee, Swiss) and platinum glass electrode. About 10 mL of the supernatant was reserved to perform the concentration analysis for Eu by using the inductively coupled plasma optical emission spectrometry (ICP-OES, iCAP 7000, Thermo, Bremen, Germany); the pH and Eh changes were also recorded simultaneously.

The sorption behavior of Eu under different conditions was shown, and the distribution coefficient *K_d_* (mL/g) was calculated using the following equations:

(1) $$K_{d}=\frac{Q}{C}=\frac{\left(C_{0}-C\right)}{C}•\frac{V}{M}$$

where *C*₀ is the initial concentration in solution, *Q* is the Eu sorbed in a solid amount (m mol/g), *C* is the Eu concentration (ppm) after the 7-day batch test, *V* is the volume (mL) of the liquid, and *M* is the weight (g) of the solid phase (the sample mixture).

### 2.3. Samples for XAS (X-ray Absorption Spectra) at NSRRC

ASTM batch sorption tests were also applied and followed in this work. Three portions of the testing rock were prepared in 50 mL centrifuge tubes for triplicate experiments. The Eu_2_O_3_ stock solution (C₀ = 0.01 M) contained stable isotope tracers and was added to deionized water with 1 M HNO_3_ prior to batch tests. All batch tests were conducted with a solid/liquid ratio of 1 g/50 mL. After 24 h of shaking, the tubes were removed and centrifuged in rpm (=10,380× *g*) for 15 min. Finally, the remaining tubes were emptied of all liquid; air-dried in a container overnight; and the solids were taken out, ground, and sieved through a 200-mesh sieve (<0.074 mm) for X-ray Absorption Spectroscopy (XAS, BL16A, NSRRC, Hsinchu, Taiwan) at the National Synchrotron Radiation Research Center (NSRRC) in Taiwan. All spectra were collected in fluorescence mode at room temperature using argon-filled ionization chambers (Lytle detector). The theoretical modeling code IFEFFIT [13] was used to analyze the X-ray absorption near-edge structure (XANES) data. The studies demonstrated the phenomena that the hydrated Eu ion can be adsorbed at planar sites or interlayer sites to form inner-sphere (IS) and outer-sphere (OS) complexes. In addition to XANES, we utilized a comprehensive dual approach to fit the extended X-ray absorption fine structure (EXAFS) spectra on the Eu surface complexation.

### 2.4. Wavelet Transform

In addition to the commonly used Fourier transform method for analyzing EXAFS, the wavelet transform is also employed in the analysis. Fourier analysis allows us to understand the composition of the first coordination shell (e.g., oxygen atoms), but it is challenging to analyze multiple scattering paths. The Morlet wavelet can help differentiate the contributions of different atoms at similar distances, distinguishing between slowly varying amplitudes and rapidly changing phases, which resemble EXAFS signals [14,15]. By utilizing the 2D distribution of EXAFS signals in K-space and R-space, contributions from multiple scattering paths can be more effectively separated. Through visual distribution, the presence of neighboring atoms in specific regions was observed, followed by EXAFS fitting analysis to determine the atomic structure information. The formula for the Morlet wavelet is as follows:

(2) $$\psi \left(t\right)=\frac{1}{\sqrt{2\pi }\sigma }\left(exp\left(ikt\right)-exp\left(-\frac{k^{2}}{2}\right)\right)exp\left(-\frac{t^{2}}{2\sigma^{2}}\right)$$

This study used the open-source software (ESRF’s Hama Fortran Version 3.7) for wavelet transform analysis [16], where the k and σ parameters are set to 10 and 1, respectively, to achieve appropriate k-axis resolution. k represents the frequency of sine and cosine functions. It is a free wavelet parameter indicating how many oscillations of sine waves are covered by a Gaussian envelope with a half-width of σ = 1.

## 3. Results and Discussion

### 3.1. Distribution Coefficients (K_d_) from Batch Tests

According to previous studies [4,17,18], Eu sorption on rocks is a fast-uptake reaction and reaches equilibrium within 24 h. After 7 days, the pH of Eu on rocks was recorded and ranged from approximately 5.86 ± 0.03 to 8.01 ± 0.08, and showed an Eh within the 210–300 mV range in various rock concentrations. Table 2 shows that similar batch test results indicate that *K*_d_ in argillite and basalt is higher than that in biotite and granite. In fact, the comparison among argillite, basalt, granite, and biotite shows that the order variance of distribution coefficients (*K*_d_) in basalt and argillite (2472 ± 38 to 4413 ± 69 mL/g) is greater than that in biotite and granite. This indicates their adsorption effectiveness. Therefore, this demonstrates that the major influence on sorption of Eu in basalt and argillite is iron–magnesium (Fe-Mg) oxides and clay minerals, i.e., 13.86% Fe_2_O_3_ in basalt, illite, and kaolinite in argillite [19,20,21,22,23]. The main adsorption mechanism in this study is surface complexation. Thus, argillite, which has a higher pH and iron content, exhibits a higher *K*_d_ value. Although basalt has a higher iron content, its lower pH compared to argillite may result in a lower *K*_d_ value. The lower *K*_d_ value for biotite may be due to its higher K_2_O content, which causes competitive effects between K⁺ and Cu³⁺, leading to a decrease in its *K*_d_ value.

**Figure 1:**
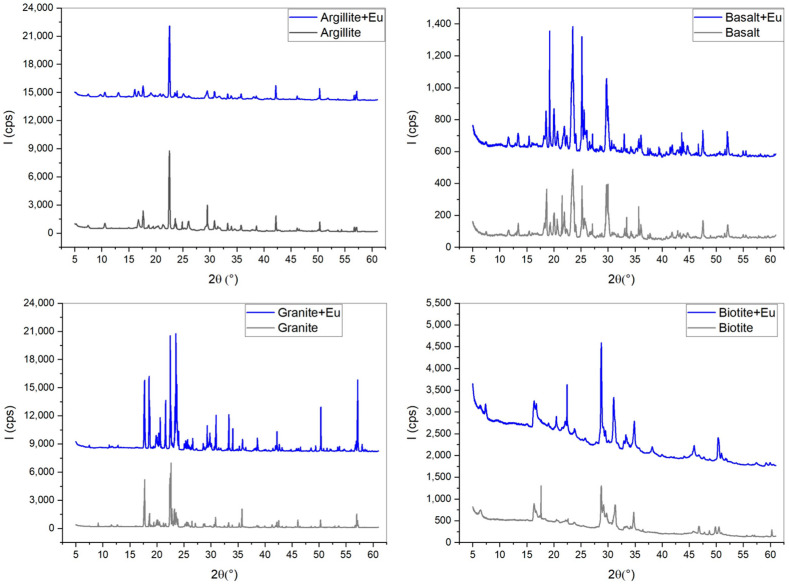
XRD for Eu adsorbed to materials.

XRD patterns in Figure 1 were identified via comparison with mineral standards, and the data were processed by employing an International Center for Diffraction Data (JPCDS) database. The results before and after Eu adsorption experiments reveal the state of Eu adsorption on the materials. Through the peak positions before and after adsorption, no significant differences are observed aside from intensity, indicating that Eu forms inner-sphere surface complexes with the material surface. When compared with *K*_d_ values, notable differences are evident in basalt, granite, and biotite, while argillite shows a decrease in the peak around $30^{{}^{\circ}}$, with no other noticeable differences.

### 3.2. XANES for Samples and Materials

**Figure 2:**
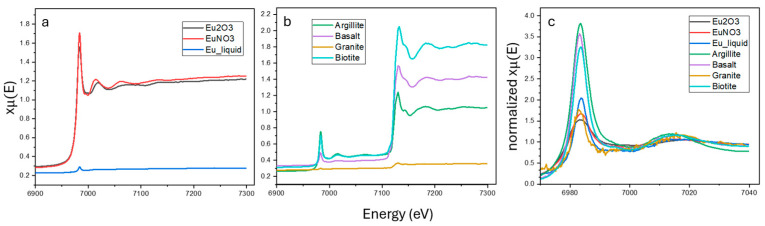
Summary of the $L_{III}$-edge XANES spectrum for Eu adsorbed onto Eu_2_O_3_, EuNO_3,_ and Eu liquid (**a**), argillite, basalt, granite, and biotite, (**b**) and normalized intensity spectrum (**c**).

Figure 2 summarizes the X-ray absorption spectrum near-edge structure (XANES) spectra for Eu adsorbed onto the standard samples (a), geological materials (b), and normalized spectral comparison curve (c). In Figure 2a,b, a prominent Eu $L_{III}$-edge absorption edge is observed at 6976.9 eV. Additionally, except for the standard samples, a noticeable second absorption edge around 7129 eV is observed, which is influenced by the Fe K-edge.

In Figure 2a, between 7000 and 7100 eV, Eu_2_O_3_ and EuNO_3_ show intensity variations in the spectra, indicating different surface complexation mechanisms for hydroxyl and nitrate groups. Additionally, in Figure 2b, argillite, basalt, granite, and biotite all exhibit adsorption reactions with Eu. However, granite shows no significant changes in spectral intensity, which is related to the *K*_d_ values in Table 2. The adsorption experiments are consistent with the XANES results. Moreover, the signal intensity, which correlates with the Fe_2_O_3_ content in XRF analysis, was observed in descending order in Table 1: basalt (13.86%), argillite (6.74%), and granite (1.09%). This is consistent with the XRF measurement results in Table 1.

In Figure 2c, a comparison of the XANES spectra reveals that none of the standard samples completely match the geological materials, which could be related to their surface complexation mechanisms. Significant changes can also be observed in the argillite spectra between 7020 and 7040 eV, which might be related to the interactions between the Eu central atom and metal ions. Further EXAFS analysis will be conducted to investigate the detailed atomic structure.

### 3.3. EXAFS for Eu Samples

**Figure 3:**
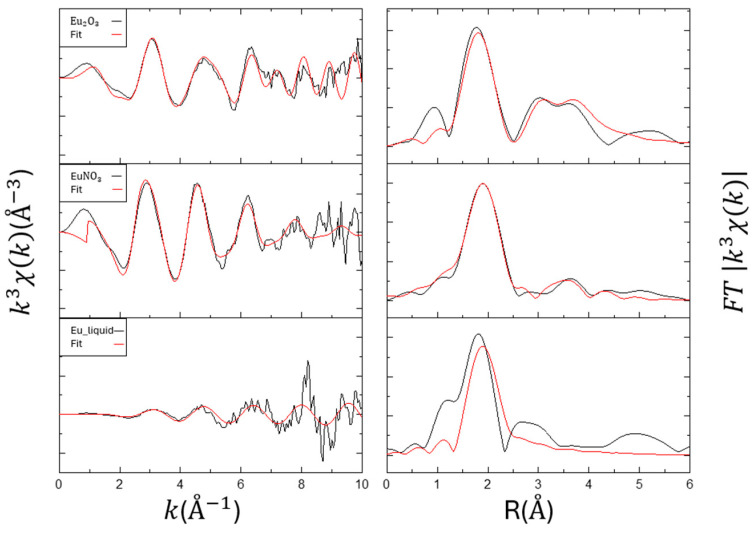
Summary of the Eu $L_{III}$-edge EXAFS and the respective Fourier transform of the adsorption samples. Left: $k^{3}$-weighted χ(k) spectra. Right: corresponding Fourier transformed spectra.

The EXAFS analysis results for the samples Eu_2_O_3_, EuNO_3,_ and Eu liquid are shown in Figure 3 and Table 3. For the standard sample Eu_2_O_3_, the first peak appears around 1.8 Å, corresponding to the Eu-O coordination in the first shell. In the R space between 3 and 4 Å, two distinct peaks are observed, indicating the influence of the second shell Eu-Eu and Eu-O interactions. Similar peaks can also be seen in [24]. The Eu-O distance is 2.36 Å, the Eu-Eu distance is 3.69 Å, and the second-shell Eu-O distance is 4.27 Å, which are consistent with experimental results from [24,25,26]. EuNO_3_ has an Eu-O distance of 2.45 Å with a coordination number of 5.0. In the R space at 3.5 Å, there is a distinct single peak, and the Eu-Eu fitting yields a distance of 4.18Å with a coordination number of 5.0. Due to being in an aqueous solution, the signals for Eu liquid are not distinct in both XANES and EXAFS analysis. Therefore, only the first shell fitting was performed, showing an Eu-O distance of 2.39 Å with a coordination number of 4.03. Additionally, the Eu-O hydration bond length is typically greater than 2.43 Å with a coordination number of 8, while the bond length on material surfaces is usually less than 2.35 Å with a coordination number of 6. The single scattering in the first shell is indeed contributed by Eu-O, consistent with previous research [27].

### 3.4. EXAFS for Materials (Effect of Fe)

**Figure 4:**
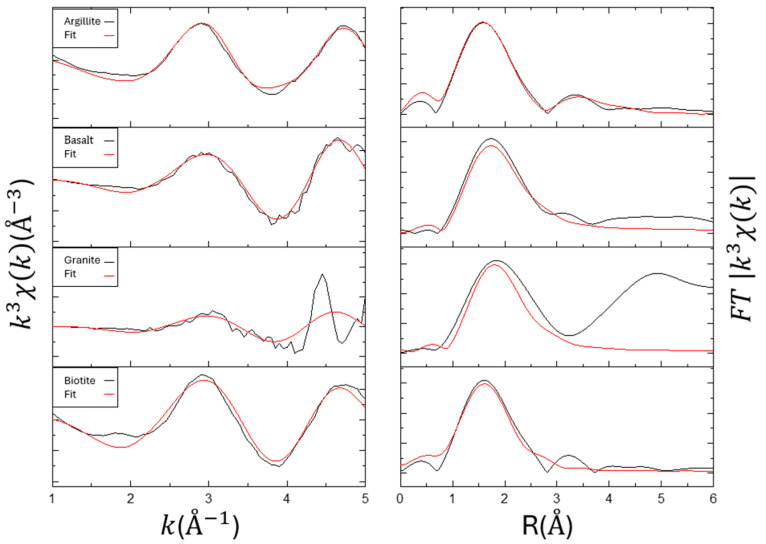
Summary of the Eu $L_{III}$-edge EXAFS and the respective Fourier transform of the adsorption with Fe. Left: $k^{3}$-weighted χ(k) spectra. Right: corresponding Fourier transformed spectra.

The EXAFS analysis employed a stepwise fitting approach. Initially, Eu-O was fitted within an R range of 1 and 3 Å results, as shown in Figure 4 and Table 4. After fixing the current results, metal ions were analyzed within an R range of 1–4 Å. The EXAFS analysis results for the materials and oxides are shown in Table 3. By using the stepwise fitting method, we were able to determine that the Eu-O contributions come from the first layer (outer-sphere layer, i.e., hydrated complexes) or the second layer (inner-sphere layer) [28,29,30].

The fitting results indicate that the outer Eu-O distance ranges from 2.42 to 2.52 Å, with a coordination number of around 2 to 3. The inner Eu-O distance is 2.27 and 2.32 Å, with a coordination number of 4 and 9. By calculating the coordination number (CN) ratio of the inner/outer layers, the results indicate that the adsorption sites are closer to the mineral’s inner layer. Excluding the case of biotite, it is observed that the *K*_d_ is related to the CN ratio. The higher the pH, the more pronounced the surface complexation effect, which reflects the adsorption mechanism of Eu surface complexation [8,11].

Through stepwise fitting of the second-shell results, the Eu-Fe distance is between 3.16 and 3.66 Å, with a coordination number of 0.82 to 4.0. It can be observed that the closer the adsorption occurs to the inner layer, indicated by a higher CN ratio, the higher the coordination number of Fe. This correlates with the order of intensity of the Fe K-edge absorption observed in XANES.

### 3.5. EXAFS for Materials (Effect of Si)

**Figure 5:**
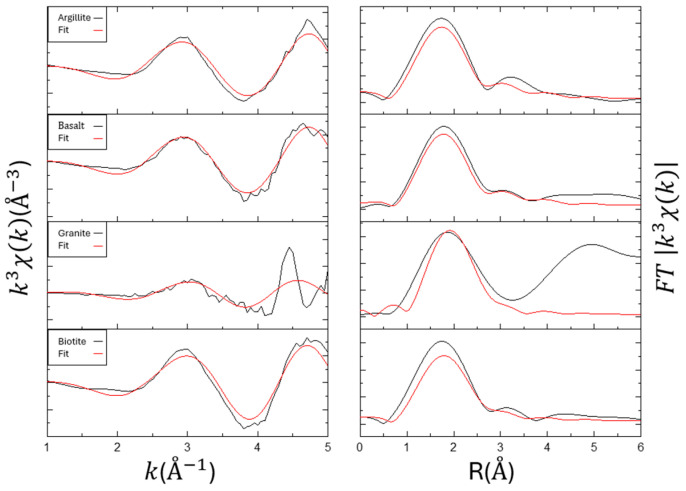
Summary of the Eu $L_{III}$-edge EXAFS and the respective Fourier transform of the adsorption with Si.

The EXAFS analysis results of materials with Eu-Si [31], shown in Figure 5 and Table 5, indicate that argillite and basalt have similar fitting results for the outer and inner layer Eu-O distances and coordination numbers, as well as Eu-Si distances. The similar CN ratios show that both materials utilize a similar mechanism for outer layer adsorption (ion exchange). Argillite has a higher Eu-Si coordination number of 6.88 compared to basalt’s 4.22, which correlates with the higher SiO_2_ content in argillite.

Granite and biotite, however, exhibit different CN ratios compared to argillite and basalt, indicating that their adsorption mechanisms are closer to the mineral’s inner layer. Although granite is rich in SiO_2_, its low adsorption capacity, reflected in the lower pH and *K*_d_ value, affects its adsorption behavior.

The CN ratios in Table 3 and Table 4 also reveal that the inner layer adsorption mechanism of Eu in argillite and basalt is primarily dominated by the Fe_2_O_3_ content. In the comparison of fitting result curves in Figure 4 and Figure 5, it can also be observed that in Figure 5, basalt shows a distinct peak around 3.1 Å. This is due to different adsorption mechanisms, leading to a greater distance between Eu and Fe/Si atoms. Wavelet transforms can more clearly reveal the differences between them.

### 3.6. Wavelet of EXAFS Fitting Results

**Figure 6:**
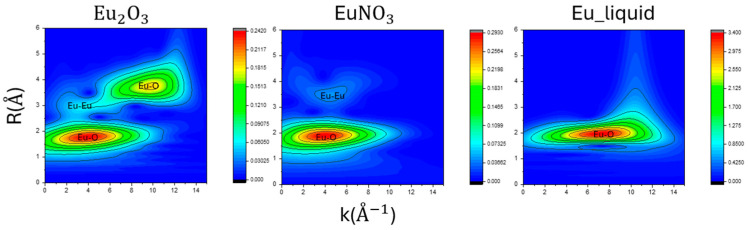
The two-dimensional spectrum of the standard sample after Morlet wavelet transformation. The spectrum is plotted with K-space, R-space, and the intensity representing the frequency content at different time points.

Applying a wavelet transform to the EXAFS data allows for visualization of the two-dimensional information. In the standard sample results shown in Figure 6, the R-space displays scattering contributions from Eu-O around an R distance of 2. The Eu liquid sample exhibits differences in the K-space compared to the other two samples, particularly in the coverage of the first peak, indicating distinctions between the solid and liquid states in the wavelet analysis results.

**Figure 7:**
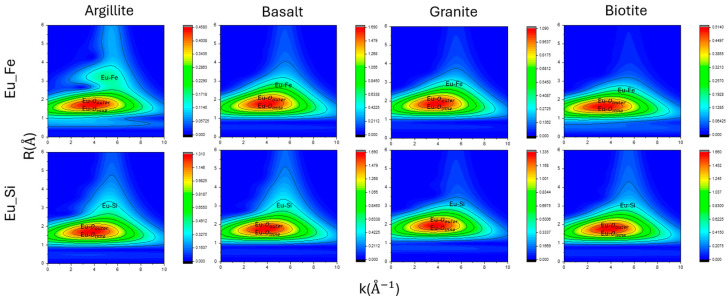
The two-dimensional spectrum of the materials after Morlet wavelet transformation. The spectrum is plotted with K-space, R-space, and the intensity representing the frequency content at different time points.

In the material results shown in Figure 7, after applying a wavelet transform to the fitted data and comparing it with the standard sample, it is observed that the size of the first peak’s red circle in the R-space is significantly larger than that of the standard sample. The K-space results, all within the range of 2–6, indicate that the first peak of Eu-O is contributed by both the inner and outer layers, aligning with our EXAFS analysis results.

In each figure, a prominent point can be seen around the R-axis in 2–4 Å and the K-axis in 4–8$\;{Å}^{-1}$, which shows the presence of other atomic scattering paths in this region. The larger the distribution coefficient (*K*_d_), such as in argillite and basalt, the more pronounced these features appear in the wavelet transform images. This corresponds to the Fe and Si atoms fitted in our EXAFS analysis.

The similarity in the wavelet transform results for Eu-Fe fitting indicates similar adsorption mechanisms, while the Eu-Si fitting for argillite and basalt, as well as granite and biotite, showing similar patterns in the prominent points, reflects the dominance of either inner or outer layer adsorption mechanisms, as indicated in the EXAFS analysis.

## 4. Conclusions

This study explored the relationship between distribution coefficients (*K*_d_) and Eu adsorption experiments. The adsorption mechanism is primarily dominated by surface complexation, with the order being basalt, argillite, and granite. Additionally, it discusses in detail the influence of Fe in the adsorption process. The adsorption experiments revealed noticeable differences in the XRD patterns of the materials before and after adsorption, while the intensity of the Fe K-edge absorption in XANES was closely related to the Fe_2_O_3_ content in the materials and the *K*_d_ values. EXAFS analysis of Fe and Si indicated that argillite and basalt have different Eu-O CN ratios, as further evidenced by the distinct differences observed in the wavelet 2D images. This shows that argillite and basalt employ different adsorption mechanisms for Fe and Si, with Fe influencing the Eu adsorption position, bringing it closer to the mineral’s inner layer. By combining EXAFS analysis with Wavelet transforms, not only can the contributions of Eu-O in the inner and outer layers be better understood, but fitting second-layer cation metals can also provide additional insights.

## Figures and Tables

**Table 1:** Elemental composition of several rocks powder by XRF analysis.

	Element	SiO_2_	Al_2_O_2_	Fe_2_O_3_	CaO	Na_2_O	K_2_O	MnO	MgO	TiO_2_	P_2_O_2_	* L.O.I
Rocks												
Argillite		65.99	15.97	6.74	0.99	1.26	2.37	0.09	2.03	0.87	0.17	5.39
Basalt		47.90	15.34	13.86	7.56	3.90	1.62	0.16	6.25	2.71	0.83	1.62
Granite		77.08	12.44	1.09	1.16	3.53	3.06	0.03	0.16	0.06	0.01	0.19

* L.O.I: Loss on Ignition.

**Table 2:** The pH, Eh, and distribution coefficients (*K*_d_) of Eu in different rock samples.

Item	Rock Samples			
	Basalt	Argillite	Granite	Biotite
pH	7.54 ± 0.02	8.01 ± 0.08	5.86 ± 0.03	8.73 ± 0.07
Eh (mV)	214 ± 11	238 ± 4	299 ± 13	282 ± 39
Q (m mol/g)	2.76 ± 0.01	4.10 ± 0.03	0.48 ± 0.10	0.89 ± 0.02
*K*_d_ (mL/g)	2472 ± 38	4413 ± 69	6.16 ± 0.19	55.20 ± 8.19

**Table 3:** Local structure of Eu(III) adsorbed on samples determined using Eu L3-edge EXAFS.

Sample	Item	R(Å)	CN	∆E_0_	R_f_ (%)
Eu_2_O_3__solid	Eu-O	2.36	4.00	0.79	2.14
	Eu-Eu Eu-O	3.69 4.27	3.41 2.00		
EuNO_3__solid	Eu-O	2.45	5.00	3.33	1.50
	Eu-Eu	4.18	5.00		
Eu_liquid_0.5M	Eu-O	2.39	4.03	3.13	21.88

R, interatomic distance; CN: coordination number; ∆E_0_, threshold E_0_ shift; R_f_, residual factor; Eu_liquid_0.5M: 1 mole of Eu_2_O_3_ is dissolved in a 1 M HNO_3_ solution.

**Table 4:** Local structure of Eu(III) adsorbed on materials determined using Eu $L_{III}$-edge EXAFS with Fe.

	First Shell (Eu–O1st)		Second Shell (Eu–O2nd/Fe)					
Sample	R(Å)	CN	Shell	R(Å)	CN	∆E_0_	R_f_(%)	CN2nd/CN_1st_
Argillite	2.42	2.54	Eu-O Eu-Fe	2.27 3.66	6.68 3.00	−3.97	1.31	2.63
Basalt	2.50	1.95	Eu-O Eu-Fe	2.31 3.17	7.03 5.47	−2.99	2.91	3.61
Granite	2.52	2.06	Eu-O Eu-Fe	2.32 3.24	4.00 0.82	−0.22	16.73	1.94
Biotite	2.48	2.91	Eu-O Eu-Fe	2.28 3.16	8.90 3.34	−4.05	5.23	3.06

R, interatomic distance; CN: coordination number; ∆E_0_, threshold E_0_ shift; R_f_, residual factor.

**Table 5:** Local structure of Eu(III) adsorbed on materials determined using Eu $L_{III}$-edge EXAFS with Si.

	First Shell (Eu–O1st)		Second Shell (Eu–O2nd/Si)					
Sample	R(Å)	CN	Shell	R(Å)	CN	∆E_0_	R_f_ (%)	CN2nd/CN_1st_
Argillite	2.42	7.00	Eu-O Eu-Si	2.20 3.85	2.23 6.88	−1.66	6.71	0.32
Basalt	2.43	7.30	Eu-O Eu-Si	2.21 3.84	2.15 4.22	−0.65	4.56	0.29
Granite	2.58	2.29	Eu-O Eu-Si	2.41 2.96	5.86 1.81	3.09	26.43	2.56
Biotite	2.48	3.50	Eu-O Eu-Si	2.31 3.41	7.00 1.31	−1.72	10.86	2.00

R, interatomic distance; CN: coordination number; ∆E_0_, threshold E_0_ shift; R_f_, residual factor.

## Data Availability

Dataset available on request from the authors.
